# Targeted NGS gene panel identifies mutations in *RSPH1* causing primary ciliary dyskinesia and a common mechanism for ciliary central pair agenesis due to radial spoke defects

**DOI:** 10.1093/hmg/ddu046

**Published:** 2014-02-11

**Authors:** Alexandros Onoufriadis, Amelia Shoemark, Miriam Schmidts, Mitali Patel, Gina Jimenez, Hui Liu, Biju Thomas, Mellisa Dixon, Robert A. Hirst, Andrew Rutman, Thomas Burgoyne, Christopher Williams, Juliet Scully, Florence Bolard, Jean-Jacques Lafitte, Philip L. Beales, Claire Hogg, Pinfen Yang, Eddie M.K. Chung, Richard D. Emes, Christopher O'Callaghan, UK10K, Patrice Bouvagnet, Hannah M. Mitchison

**Affiliations:** 1 Molecular Medicine Unit and Birth Defects Research Centre, Institute of Child Health, University College London, London WC1N 1EH, UK; 2 Present address: Department of Medical and Molecular Genetics, Division of Genetics and Molecular Medicine, King's College London School of Medicine, Guy's Hospital, London SE1 9RT, UK; 3 Department of Paediatric Respiratory Medicine, Royal Brompton and Harefield NHS Trust, Sydney Street, London SW3 6NP, UK; 4 Laboratoire Cardiogénétique, Equipe d'Accueil 4173, Université Lyon 1, Hôpital Nord-Ouest, Villefranche sur Saône, Lyon, France; 5 Laboratoire Cardiogénétique, Hospices Civils de Lyon, Groupe Hospitalier Est, 69677 Bron, France; 6 Department of Infection, Immunity and Inflammation, Division of Child Health, CSB, University of Leicester, LeicesterLE2 7LX, UK; 7 Institute of Ophthalmology, University College London, London EC1V 9EL, UK; 8 Service de Pneumologie, Centre Hospitalier Régional de Roubaix, Hôpital Victor Provo, Roubaix, France; 9 Département de Pneumologie, Centre Hospitalier Régional Universitaire de Lille, Hôpital Albert Calmette, Université Lille 2, Lille, France; 10 Department of Biology, Marquette University, Milwaukee, WI 53233, USA; 11 General and Adolescent Paediatric Unit, Institute of Child Health, University College London, London, UK; 12 School of Veterinary Medicine and Science, University of Nottingham, Leicestershire LE12 5RD, UK; 13 Advanced Data Analysis Centre, University of Nottingham, Sutton Bonington Campus, Leicestershire LE12 5RD, UK; 14 Department of Respiratory Medicine, Portex Unit, Institute of Child Health, University College London and Great Ormond Street Hospital, 30 Guilford Street, LondonWC1N 1EH, UK; 15 uk10k.org.uk and; 16 Service de Cardiologie Pédiatrique, Hospices Civils de Lyon, Groupe Hospitalier Est, 69677 Bron, France

## Abstract

Primary ciliary dyskinesia (PCD) is an inherited chronic respiratory obstructive disease with randomized body laterality and infertility, resulting from cilia and sperm dysmotility. PCD is characterized by clinical variability and extensive genetic heterogeneity, associated with different cilia ultrastructural defects and mutations identified in >20 genes. Next generation sequencing (NGS) technologies therefore present a promising approach for genetic diagnosis which is not yet in routine use. We developed a targeted panel-based NGS pipeline to identify mutations by sequencing of selected candidate genes in 70 genetically undefined PCD patients. This detected loss-of-function *RSPH1* mutations in four individuals with isolated central pair (CP) agenesis and normal body laterality, from two unrelated families. Ultrastructural analysis in *RSPH1*-mutated cilia revealed transposition of peripheral outer microtubules into the ‘empty’ CP space, accompanied by a distinctive intermittent loss of the central pair microtubules. We find that mutations in *RSPH1*, *RSPH4A* and *RSPH9,* which all encode homologs of components of the ‘head’ structure of ciliary radial spoke complexes identified in *Chlamydomonas*, cause clinical phenotypes that appear to be indistinguishable except at the gene level. By high-resolution immunofluorescence we identified a loss of RSPH4A and RSPH9 along with RSPH1 from *RSPH1*-mutated cilia, suggesting RSPH1 mutations may result in loss of the entire spoke head structure. CP loss is seen in up to 28% of PCD cases, in whom laterality determination specified by CP-less embryonic node cilia remains undisturbed. We propose this defect could arise from instability or agenesis of the ciliary central microtubules due to loss of their normal radial spoke head tethering.

## INTRODUCTION

Cilia are complex, dynamic microtubule-based structures conserved across eukaryote species that extend as hair-like projections from the cell surface. Their growth and resorption is tightly cell cycle-linked, and their components are synthesized in the cell body then actively transported into the ciliary compartment by intraflagellar transport (1–3). Human motile cilia and flagella (sperm tails) are structurally related and play essential roles in the motility of fluid and cells. Proteins required for motile cilia functions may also have roles in cilia assembly and orientation of cell divisions (4). Motile cilia extend from the multiciliated epithelial surface of the respiratory airways, the brain ependyma and fallopian tubes (5). They beat in synchrony with a coordinated ‘whiplash’ motion, forwards and back within the same plane to achieve fluid flow across their surface, and the initiation and complex regulation of this process is under the control of a variety of intracellular and external factors (6).

The core axoneme structure of cilia and flagella contains a central pair (CP) of singlet microtubules (‘CP’) surrounded by nine doublet microtubules giving a classic ‘9 + 2’ arrangement when viewed in cross-section. Motile monocilia that initiate left–right body axis patterning during embryogenesis lack the CP apparatus in a ‘9 + 0’′ arrangement, and have a circular beat giving rise to leftward fluid flow across the embryonic node (7,8). Much of what we know about motile cilia function is derived from studies of ancient flagellate organisms such as the single-celled biflagellate green alga *Chlamydomonas reinhardtii* (9). There are a number of microtubule-associated protein complexes attached along the length of the axoneme in a regular 96 nm repeat formation that provide axonemal stability to support the rigors of ciliary beating, whilst tightly governing the velocity of the beat and the waveform pattern (10). Pairs (inner and outer) of dynein arm motor complexes attached to the peripheral doublets are responsible for ATP-generated ciliary beating which requires coordinated sliding interactions between the peripheral doublet microtubules of the axoneme. The complex regulation of ciliary motility is not fully understood, but key structural and regulatory complexes governing local dynein motor activity are the nexin–dynein regulatory complexes that link between the peripheral doublets, and the radial spoke complexes (11). Both of these structures are located in close proximity to the dynein motors within the 96 nm repeat. The radial spokes are T-shaped assemblies comprising at least 23 proteins that are composed of a head and stalk, which together form a major radial scaffold for mechanochemical signal transduction along the axoneme (12). They link between the CP outwards to the peripherally located dynein motors, directing ciliary beating waveform and velocity (13).

Primary ciliary dyskinesia (PCD; MIM244400) is a genetically heterogeneous disorder arising from abnormal motility of cilia and flagella, associated with defects of the axoneme that are usually ultrastructurally visible (14). The disease manifests with an autosomal recessive mode of inheritance and affects one in every 15 000–30 000 births. The clinical course of disease can greatly vary and abnormal cilia motility causing deficient mucociliary transport in the airways leads to a number of symptoms including chronic respiratory infections, rhinosinusitus, effusive otitis media with progression to irreversible lung damage (bronchiectasis). Dysmotile fallopian tube cilia and sperm flagella give rise to subfertility that can affect both sexes in adulthood. Dysfunction of motile embryonic node cilia arising during development gives rise to situs abnormalities in approximately half of individuals with PCD. This reflects a randomized left–right axis determination which usually manifests as clinically harmless situs inversus, however, more complex laterality defects can give rise to clinically severe isomerisms and heart defects in ∼6% of cases (15).

Genetic studies have highlighted extensive genetic heterogeneity underlying PCD within the last decade, and it is sometimes described as a group of disorders rather than single disease entity. Although the molecular basis of a significant proportion of PCD cases still remains unexplained, a number of genetically stratified patient subgroups can be identified based on ultrastructural and motility findings in their cilia. Mutations in 27 genes leading to distinct ultrastructural defects have been reported (16–22, and references therein). These include mutations in structural components of the axoneme including the outer dynein arms (ODA) and their targeting and docking complexes, causing ODA defects (*DNAH5, DNAI1, DNAI2, DNAL1, NME8/TXNDC3, CCDC114* and *ARMC4*). Some mutations affecting components or regulators of the nexin–dynein regulatory complexes cause microtubule disarrangements and inner dynein arm (IDA) loss (*CCDC39, CCDC40*) whilst others do not greatly disturb axoneme organization (*CCDC164, CCDC65*), a finding also noted for mutations affecting an ODA component gene (*DNAH11).* Mutations resulting in dual loss of the outer and IDA have also been identified in genes encoding cytoplasmic or cytoplasmic/axonemal proteins with roles in the cytoplasmic assembly and/or transport and docking of dynein arm components, (*DNAAF1/LRRC50, DNAAF2/KTU, DNAAF3, CCDC103, HEATR2*, *LRRC6, ZMYND10, DYX1C1, SPAG1, C21ORF59*). *RPGR* mutations appear to cause a syndromic form of PCD (23).

Lastly, mutations in four genes encoding components of the CP (*HYDIN*) and the radial spokes (*RSPH4A, RSPH9, RSPH1*) all cause a subset of PCD characterized by defects affecting solely the CP (i.e. CP deficiency/loss without significant disturbance to other structures). In HYDIN mutant respiratory cilia which lack only the C2b projection of the CP (24), the cilia ultrastructure in fact looks mostly undisturbed and a complete CP loss is only occasionally seen. CP loss is a much more frequently seen event in *RSPH4A, RSPH9* and *RSPH1* mutant cilia, and this makes *HYDIN* a distinct subtype defined by largely normal-looking cilia requiring electron tomography for their subtle defect to be seen (20,25). CP loss can occasionally also occur as a component of other defects, for example, as part of the general microtubular disarrangements caused by *CCDC39* and *CCDC40* mutations, but again this is a distinct subtype where CP disturbances form part of a different overall pattern of defects.

Estimates vary from country to country, but CP deficiency has been reported to account for as much as 28% of all PCD cases (26,27). Individuals with CP only-deficient PCD do not have laterality defects, and the 9 + 0 ultrastructure of the embryonic nodal cilia would not be predicted to be influenced by mutations affecting the CP which they lack (28,29). The complete loss of the CP is typically also referred to as ‘transposition’ defect, since the affected cilia often display a peripheral doublet transposed into the ‘empty’ CP area to create an aberrant ‘8 + 1’ microtubule pattern viewed in cross-sections, in addition to having abnormal ‘9 + 0’′ cross-sections (25,29).

The high level of genetic heterogeneity underlying PCD has previously led to labor-intensive and expensive genetic screening, especially, since many of the causative genes are large dyneins. An attractive approach that has not yet been widely implemented for PCD genetic diagnostics is the use of high-throughput DNA sequencing techniques offered by next generation sequencing (NGS), where multiple genes can be analyzed for mutations in a rapid and cost-efficient way. Here we used an NGS ‘gene panel’ approach to investigate the genetic basis of PCD in a cohort of genetically undefined, unrelated individuals, and demonstrate successful gene identification and sub-stratification of the CP-deficient form of PCD.

## RESULTS

### Targeted NGS sequencing identifies *RSPH1* mutations in a cohort of PCD patients

We employed an NGS approach to identify the disease-causing mutations in 70 unrelated individuals with a clinical diagnosis of PCD. Genomic DNA isolated from peripheral blood samples was subjected to NGS resequencing of a panel of selected ciliopathy genes. This included all the currently identified PCD-causing genes, as part of a larger panel containing 150 candidate PCD genes suggested from our own on-going and collaborative studies. The next generation sequence data generated was quality controlled, aligned back to the genome, and annotated for DNA variants. The total variant list generated for each individual was then filtered for consistency with a rare recessive disease model. This analysis required the presence of at least one homozygous or two heterozygous changes that were either novel or occurring with an estimated frequency <0.01 in publically available human exome databases (1000 Genomes, NHLBI EVS, dbSNP132). Based on pre-existing knowledge of PCD mutations, we focused especially on non-synonymous or splice-site substitutions and indels, and a final filter was applied to identify variants present in genes with putative function in motile cilia and/or those present in the Cilia Proteome (30).

The gene panel included the known CP loss/transposition defect-associated genes *RSPH4A, RSPH9* and *RSPH1*, as well as *HYDIN.* Two of five CP loss/transposition defect cases within the cohort (PCD-166 II:1 and PCD-282 III:1) were found to carry low-frequency biallelic variants in the 1.4 kb, 8 exon *RSPH1* cDNA sequence, which spans 24 kb on chromosome 21q22.3*.* The variants identified in the rest of the cohort are currently under analysis. The sequencing and filtering results for individuals PCD-166 II:1 and PCD-282 III:1 are shown in Table 1. Both are females with typical symptoms of PCD including neonatal respiratory distress episodes. In later life both continued to suffer chronic disease symptoms of productive cough, rhinitis and sinusitis, recurrent respiratory infections and congestive lung symptoms. Other documented signs in both cases were ear infections and otitis media, which had led to hearing problems requiring grommet surgery, and both also had bronchiectasis. Nasal nitric oxide in PCD-166 II:1 was greatly reduced consistent with other PCD patients, measuring 50 and 79 ppb in independent tests [normal range >200 ppb; (31) no data available for family PCD-282]. Two siblings in PCD-282 were also similarly affected.

**Table 1.** Summary of gene panel sequencing data filtering process

	PCD-166 II:1	PCD-282 III:1
Total variants	5064	5612
Variants with MAF < 0.01	587	669
Heterozygous, MAF < 0.01	462	514
Heterozygous non-synonymous, splice-site, or insertion/deletion variants	130	92
Genes with compound heterozygous variants	8	9
Genes with motile cilia functions	2 *DNAH12*^a^: c.5093G>A; p.Pro1698Leu & c.3577C>A; p.Ala1193Ser (rs2016874) *RSPH1*: c.281G>A; p.Trp94* & c.275-2A>C splice site (rs1511075)	2 *DNAH1*^a^: c.3103C>T; p.Arg1035Cys & c.12090 + 7A>C splice site (rs180991820) *DNAH3*^a^: c.5368T>A; p.Ile1790Phe (rs148202152) & c.7C>T; p.Ala3Thr
		
Homozygous, MAF < 0.01	125	155
Homozygous non-synonymous, splice-site or insertion/deletion variants	9	7
Genes with motile cilia functions	None	3 *RSPH*1: c.85G>T; p.Glu29* *WDR66*^b^: c.186-187ins of 15 base-pairs; p.62-63insEEEEK (rs142042908) *CCDC40*^c^: c.3040-3041insCAC; p.1014insThr (rs10693712)

^a^Dynein heavy chain mutations not expected to cause PCD with central pair (CP) loss.

^b^*WDR66* is upregulated during ciliogenesis, but not previously associated with PCD.

^c^*CCDC40* mutations cause PCD with disorganized microtubules and inner dynein arm (IDA) loss, not CP-only loss. *WDR66* and *CCDC40* were excluded by segregation analysis. MAF, minor allele frequency.

Of 17 genes in PCD-166 II:1 showing biallelic variants meeting the filtering criteria, searches for putative motile cilia function applied as a final filter showed variants of interest were present in only two genes that were implicated in motile cilia function: *DNAH12* and *RSPH1* (Table 1). Of 16 genes in PCD-282 III:1 with biallelic variants, this final filter detected biallelic variants in five genes with possible motile cilia roles: *DNAH1, DNAH3, WDR66, CCDC40* and *RSPH1* (Table 1). Since the ciliary ultrastructural defects in the two cases were not compatible with mutations in dynein arm components, this excluded the *DNAH12, DNAH1* and *DNAH3* mutations as a likely cause. *CCDC40* causes PCD with a different defect of microtubule disorganization and IDA loss (32), not seen in these cases, and *WDR66* although highly expressed in ciliated tissues has an unknown function (33). Furthermore, for the *CCDC40* and *WDR66* variants we performed segregation analysis which excluded the mutations in both as likely to be disease-causing, since unaffected individuals from PCD-282 also carried both the variants in homozygous form (data not shown).

**Figure 1.**
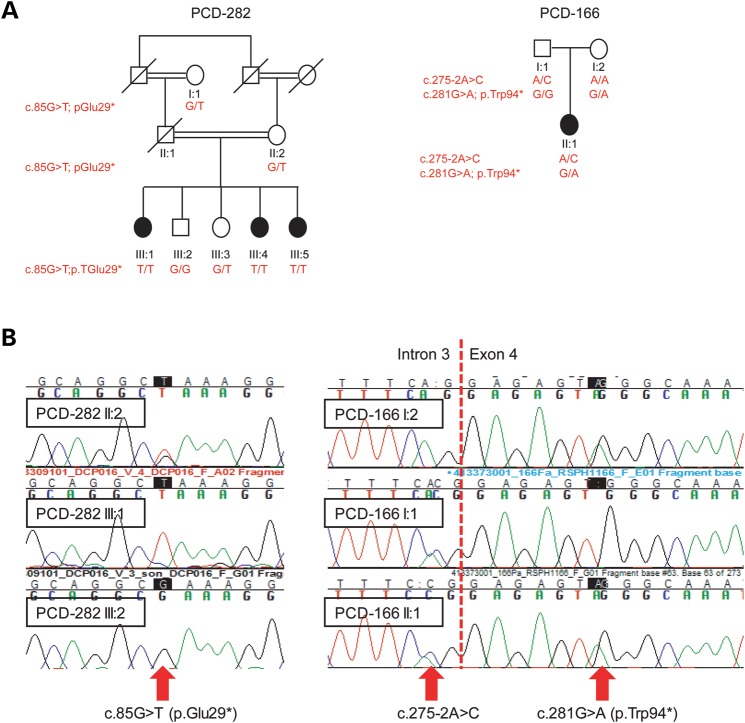
Sanger sequencing showing familial segregation of the *RSPH1* mutations. (**A**) Pedigrees of families with *RSPH1* mutations found in this study, demonstrating segregation consistent with autosomal recessive inheritance. (**B**) Sanger sequencing confirmed the segregation of three *RSPH1* mutations as indicated in the pedigrees.

Of all these putative disease-causing variants, only *RSPH1* carried nonsense mutations in the two affected individuals (Table 1). PCD-166 II:1 from a non-consanguineous Caucasian pedigree carried compound heterozygous *RSPH1* variants consisting of a splice-site change c.275-2A>C affecting one of the 100% conserved exon 4 splice acceptor bases, and a nonsense variant in exon 4, c.281G>A; p.Trp94*. PCD-282 III:1 from a consanguineous pedigree carried an early homozygous nonsense variant in exon 2, c.85G>T; p.Glu29*. All three variants are predicted to cause a major deleterious change to the RSPH1 protein, consistent with previous reporting that PCD is a ‘null’ allele disease, and they were all confirmed by Sanger sequencing (Fig. 1B). The c.275-2A>C splice change and c.85G>T nonsense allele have recently also been reported by Kott *et al*. (20) to cause PCD, whilst the identification of the c.281G>A nonsense change provides further support for *RSPH1* as a new gene which causes PCD when mutated. The unreported c.281G>A mutation is the only one of the three variants found to be completely unique, since c.85G>T is carried by 5/6,500 and c.275-2A>C by 9/6500 unaffected individuals in heterozygous state in the NHLBI EVS control exome database. However, given the expected disease and carrier frequencies for PCD, these rare, non-biallelic, occurrences in unaffected carriers are still consistent for being deleterious PCD-associated alleles.

Segregation analysis was performed in available members of the two pedigrees, and this confirmed their recessive inheritance consistent with disease status in both families (Fig. 1A and B). In total four affected individuals in both families were confirmed to carry *RSPH1* mutations, none of whom had laterality defects, which is consistent for CP loss/transposition defects (Fig. 1A). PCD-166 II:1 is too young to have information about fertility status, but notably the affected female III:1 from family PCD-282 has infertility problems. Furthermore her affected sibling, III:4 of pedigree PCD-282, was infertile and not able to conceive over 3 years despite a check-up before *in vitro* fertilization (IVF) which found no anomalies; III:4 gave birth to male twins, but only after IVF.

**Figure 2.**
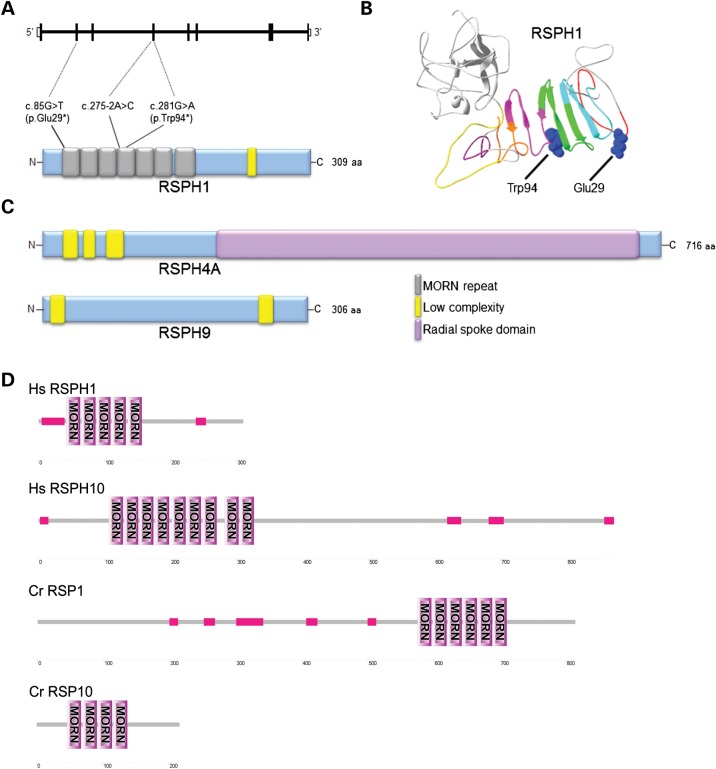
Structural models of RSPH1, RSPH4A and RSPH9 showing location of the *RSPH1* mutations. (**A**) The predicted structure of RSPH1 is shown with the position of the three identified mutations shown in relation to the gene and protein structure. RSPH1 has a low complexity region (yellow) at amino acid residues 238–251, and seven predicted MORN repeats (gray) according to Pfam and detailed in (**B**), consistent with the predicted structure of the mouse homolog (35). The Pfam consensus structure was chosen because Uniprot and SMART each predict six of these MORNs, but two are non-overlapping. (B) The three dimensional RSPH1 protein structure as predicted by I-TASSER is shown, with the seven MORN repeats highlighted at amino acid residues 26–43 (red), 44–66 (cyan), 67–89 (green), 90–112 (magenta), 113–133 (orange), 137–152 (purple) and 159–181 (yellow), in relation to the p.Trp94* and p.Glu29* nonsense mutations. (**C**) Shown in comparison to (A) are the protein structures of RSPH4A containing low complexity regions at residues 29–45, 53–66 and 80–100 (yellow) and a conserved region termed the ‘radial spoke domain’ at 207–697 (pink); and RSPH9 containing low complexity regions at residues 5–21 and 250–268. (**D**) Protein alignments of human (Hs) and *C. reinhardtii* (Cr) MORN repeat proteins of the radial spoke head show that the size of human RSPH1 (309 amino acids) is closer to CrRSP10 (216 amino acids) than CrRSP1 (814 amino acids). However, multiple sequence alignments and phylogenetic analyses support the genome annotation that RSPH1 closer resembles CrRSP1, which is 500 amino acids longer but has more similar sequences to RSPH1 outside of the MORN repeats (shown in pink). Human RSPH1 and CrRSP1 spoke head proteins therefore appear to have diverged in the sequences flanking their MORN motifs.

We modeled the identified mutations in the context of the predicted RSPH1 protein structure, to understand more about their possible mutational effect. A consensus structure defined from Uniprot, Pfam and SMART analysis identified the key domains of RSPH1 to be seven predicted MORN (membrane occupation and recognition nexus) repeats (Fig. 2A). The c.281G>A variant newly reported in this study is located very close to the c.275-2A>C splice change also affecting exon 4, and both mutations as well as c.85G>T are located in one of the seven MORN repeat regions (Fig. 2A). MORN repeats have a consensus 23 amino acid sequence and impose a predictable tertiary structure on the protein (Fig. 2B). They have been proposed to influence various essential protein functions such as membrane binding, enzymatic activity, to facilitate protein scaffold formation, and regulate a protein's accessibility to lipids (34). RSPH1 is also known as meichroacidin or TSGA2 (testis specific A2 homolog), a highly conserved axonemal protein essential for sperm flagellum formation and the production of functional sperm in mutant mice (35,36).

RSPH1 is considered to be the homolog of *C. reinhardtii* (*Cr*) *Cr*RSP1, one of the five subunits of the radial spoke head proteins that exist in this species as a multiprotein complex, whereby *Cr*RSP1 together with *Cr*RSP4, *Cr*RSP6, CrRSP9 and *Cr*RSP10 forms the radial spoke head (12,37). Human RSPH1 also has homology to *Cr*RSP10, another MORN radial spoke head protein, however *Cr*RSP10 is more diverged from RSPH1 than CrRSP1 at sequences surrounding the MORN repeats, thus *Cr*RSP1 is more likely to be the true homolog (Fig. 2D). We also compared the predicted structures of the human RSPH4A and RSPH9 proteins to human RSPH1 since they all associate in the same ciliary complex and can all carry PCD-associated mutations. However, no extensive similarities outside of the MORN repeats were apparent (Fig. 2C).

### *RSPH1* mutations cause a specific pattern of intermittent and complete ciliary CP loss, accompanied by transposition defects

**Figure 3.**
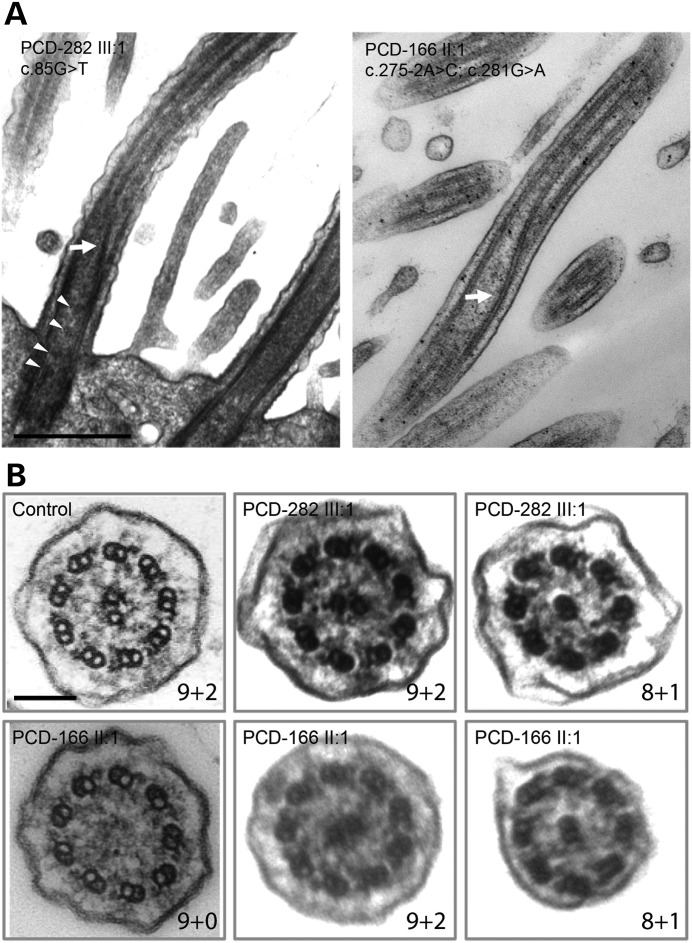
Ciliary ultrastructural defects in individuals carrying *RSPH1* mutations reveal distal central pair (CP)-deficient transposition defects sometimes retaining intermittent CP proximally. (**A**) Representative longitudinal TEM of affected individuals PCD-282 III:1 (left) and PCD-166 II:1 (right) shows the transposition event characteristic of the CP-deficient cilia. This occurs when a peripheral microtubule doublet is found in the central area of the cilium, in place of the central microtubule pair (white arrows) and this corresponds to an 8 + 1 pattern in cross-sections. Towards the cell body immediately beneath the transposition event the central area is visibly ‘empty’, lacking the CP completely, which corresponds to a 9 + 0 pattern in cross-sections. Further below, a partial CP is visible as a stump extending up from the base of the cilium, and this can sometimes be captured as having an intermittent appearance, apparently coming in and out of the plane of section (white arrow heads in PCD-282 III:1, left), however this ‘intermittent’ CP is not always visible in sections, as seen in individual PCD-166 II:1. Scale bar, 500 nm (**B**) Examples of transmission electron micrographs of cilia from the same affected individuals shown in cross section compared with control (9 + 2), showing they can have either a normal 9 + 2 arrangement, or 9 + 0, whilst more occasionally 8 + 1 sections are also seen. Scale bar, 100 nm.

Transmission electron microscopy (TEM) of respiratory cilia cross-sections from affected individuals PCD-166 II:1 and PCD-282 III:1 revealed loss of the CP apparatus with a 9 + 0 pattern in 11% and 22% of cilia in PCD-166 II:1 in two separate samples, and cross-sections with 8 + 1 transposition events more rarely seen (Fig. 3B). PCD-282 III:1 showed similar results with 30% of cross sections having the 9 + 0 pattern of absent CP, while 4% had 8 + 1 transposition events (Fig. 3B). The ciliary beat frequency and beat pattern of *RSPH1*-mutated cilia was measured and for both PCD-166 II:1 and PCD-282 III:1 the mean CBF at 37°C was in the normal 7.5–15.5 Hz range (28), measuring 11.7 and 11.4 Hz, respectively. This normal level of beating is consistent with the intact retention of the inner and ODA that is seen in TEM of the cilia from both cases. However, despite normal range frequency, a mixed beat waveform consisting of mostly stiff, unbending and uncoordinated cilia of reduced amplitude was evident for both patients, when viewed from the side (Supplementary Material, Videos S1, S3, S5). Viewed from above, regions with a circular beating pattern instead of the normal forward and backward planar motion were in both patients (Supplementary Material, Videos S2, S4). This apparently circular appearance of the beating pattern is characteristic of ciliary CP loss/ transposition defects previously reported, and it has been considered to be reminiscent of CP less 9 + 0 nodal monocilia (25,28).

From these results we concluded that the frequency of 9 + 2, 9 + 0 and 8 + 1 ultrastuctural defects in individuals carrying *RSPH1* mutations is similar to that of individuals we previously reported with mutations in *RSPH9* (25), with the CP absent in 20% of cross-sections (9 + 0) and transposition events (8 + 1) captured in < 5%. We examined longitudinal sections of *RSPH1*-mutated cilia in TEM to understand the reasons for this more clearly. We were able to image transposition events in both individuals PCD-166 II:1 and PCD-282 III:1, whereby one peripheral doublet was transposed to the apparently empty space left in absence of a CP (Fig. 3A, white arrows). These transposition events tended to be towards the distal end of the cilia, corresponding to 8 + 1 cross sections. Immediately below, i.e. towards the cell body, the axonemes completely lacked the CP, corresponding to 9 + 0 cross sections. Even further towards the cell body and the basal body, an apparently remnant CP was intermittently visible in some images that captured cilia in longitudinal section (Fig. 3A, arrow heads). This intermittent CP pattern is similar to that of individuals we previously reported with mutations in *RSPH9* (25).

### *RSPH1* mutations cause specific loss of the radial spoke ‘head’ domains from cilia, leaving the radial spoke ‘stalk’ domains intact

**Figure 4.**
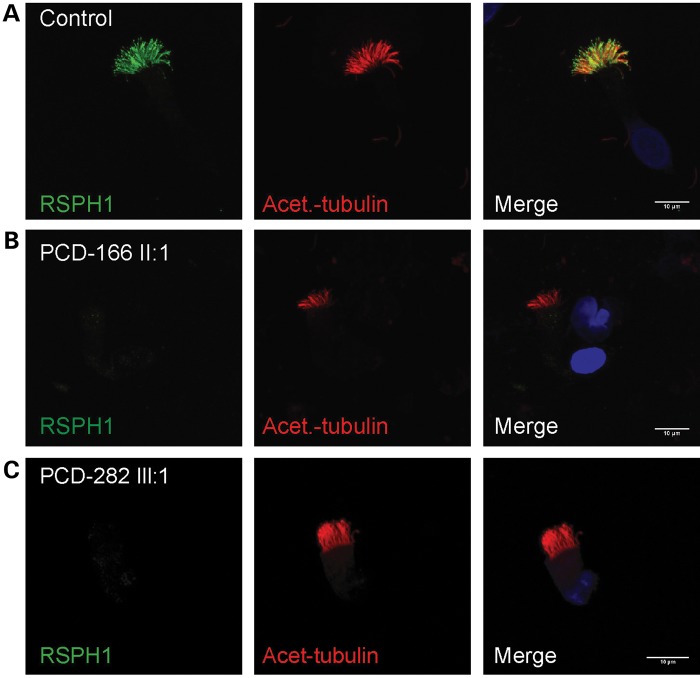
Individuals carrying *RSPH1* mutations lack RSPH1 protein along the length of the axoneme. High-resolution immunofluorescence analysis in respiratory epithelial cells obtained by nasal biopsy is shown in unaffected controls compared with individuals carrying mutations in *RSPH1* with anti-acetylated-α-tubulin used as a marker to stain the entire axoneme (red) compared with anti-RSPH1 (green), with nuclei DAPI-stained to show the DNA (blue). (**A**) RSPH1 protein is detected along the length of the cilia in a healthy individual. Individuals PCD-166 II:1 (**B**) and PCD-282 III:1 (**C**) have markedly reduced levels of RSPH1 protein. Scale bars represent 10 μm.

In order to further understand the genetic basis of the CP loss/transposition defect in PCD, in particular the connection to radial spoke head deficiency, we first analyzed the subcellular localization of RSPH1 using high-resolution immunofluorescence microscopy in ciliated epithelial cells. For this and subsequent immunofluorescence analysis, comparative images were taken with the same settings on the microscope to attempt to directly assess differences in fluorescence intensity. Whilst RSPH1 protein decorated the entire axonemal length in cilia from a control individual (Fig. 4A), reduced levels were observed along the length of cilia in both PCD-166 II:1 and PCD-282 III:1 when compared with control individuals (Fig. 4B and C). This provides confirmatory evidence that the mutations identified in the two families are disease-causing changes deleterious to protein function as predicted.

**Figure 5.**
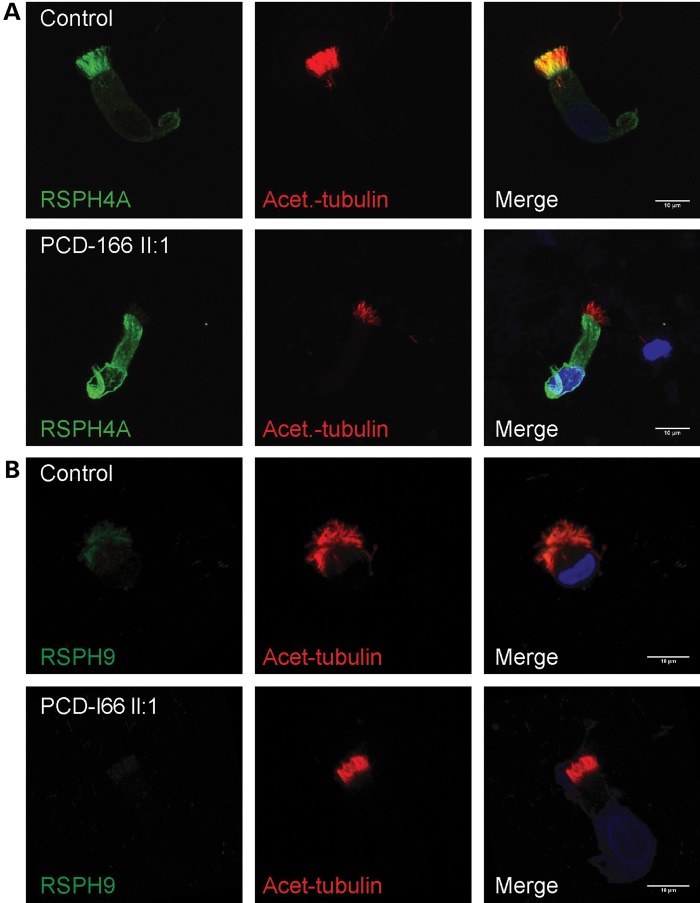
Individuals carrying *RSPH1* mutations lack other proteins of the radial spoke head suggesting the entire structure may be absent. High-resolution immunofluorescence analysis in respiratory epithelial cells obtained by nasal biopsy is shown in unaffected controls compared with individuals carrying mutations in *RSPH1* with anti-acetylated-α-tubulin used as a marker to stain the entire axoneme (red) compared with two markers of the radial spoke head complex that are also implicated in PCD, anti-RSPH4A and anti-RSPH9 (both green). Nuclei are DAPI-stained to show the DNA (blue). (**A**) RSPH4A and (**B**) RSPH9 proteins decorate the entire cilia in healthy individuals whilst levels of both proteins in PCD-166 II:1 are severely reduced. Scale bars represent 10 μm.

**Figure 6.**
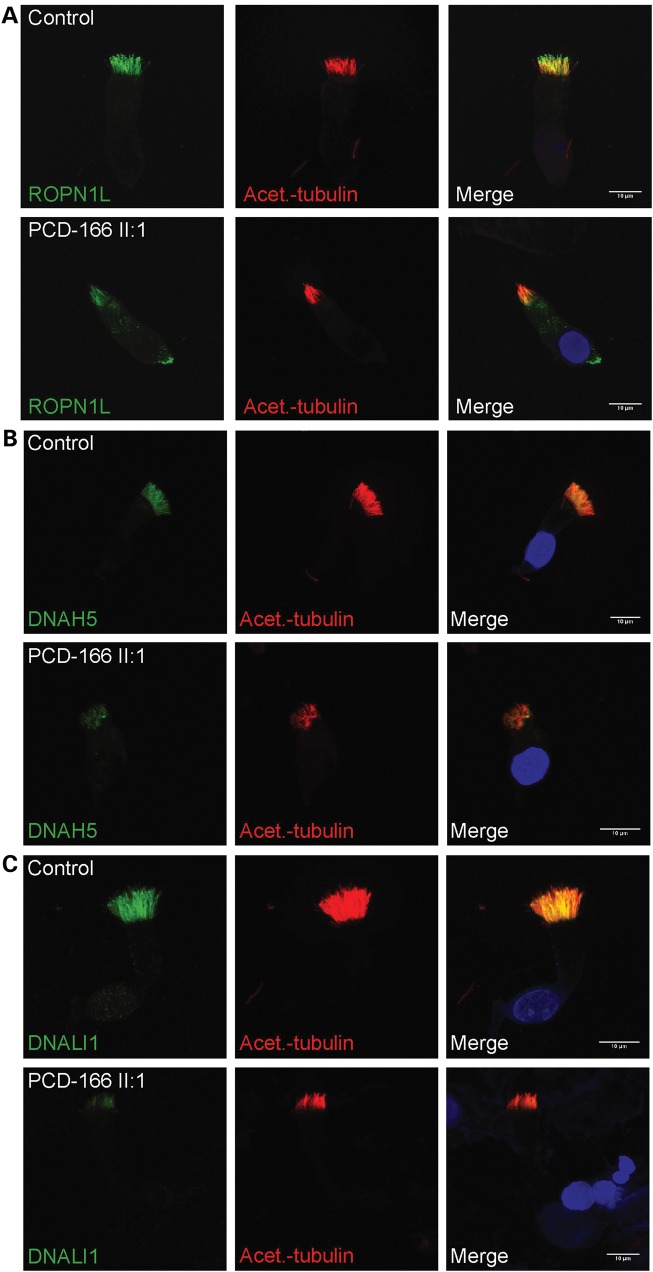
Individuals carrying *RSPH1* mutations retain markers of the radial spoke stalk and the inner and outer dynein arms (ODA). High-resolution immunofluorescence analysis in respiratory epithelial cells obtained by nasal biopsy is shown in unaffected controls compared with individuals carrying mutations in *RSPH1* with anti-acetylated-α-tubulin used as a marker to stain the entire axoneme (red) compared with a marker of the radial spoke stalk complex, anti-ROPN1L (green) and antisera against the ODA protein DNAH5 and the inner dynein arm (IDA) protein DNALI1 (green). Nuclei are DAPI-stained to show the DNA (blue). (**A**) ROPN1L, (**B**) DNAH5 and (**C**) DNALI1 immunostaining in PCD-166 II:1 is unaltered compared with healthy individuals. Scale bars represent 10 μm.

We then used antibodies targeting proteins localized in either the head domain (RSPH4A and RSPH9, both implicated in PCD), or the stalk domain (ROPN1L) of the radial spokes. RSPH4A and RSPH9 are homologs of the *Chlamydomonas* spoke head proteins RSP4 and RSP9 (12). ROPN1L, a ropporin family protein, is the homolog of *Chlamydomonas* RSP11 colocalizing with RSP3 towards the base of the radial spoke within the radial spoke ‘stalk’, and ROPN1L-deficient mice display sperm dysmotility (38,39). This immunofluorescence analysis revealed a marked reduction in levels of both RSPH4A and RSPH9 spoke head markers from the ciliary axonemes of individuals carrying *RSPH1* mutations compared with controls (Fig. 5A and B). In addition, an increased staining of RSPH4A was noted in the periciliary region and cytoplasm, suggesting the possibility that an accumulation of this protein in these compartments occurs as a result of RSPH1 deficiency. In contrast, the spoke stalk marker ROPN1L remained largely unaffected in *RSPH1*-mutated cilia, with staining similar to that of control cilia (Fig. 6A). This evidence supports the proposed role for RSPH1 in the human radial head spoke formation, and suggests that deficiency of RSPH1 affects only the spoke head structure but leaves the spoke stalks intact. This is similar to findings in *Chlamydomonas* mutant strains lacking radial spoke head proteins, in which the radial spoke stalk complex is not disturbed (12,37). In this study, the mislocalized RSPH4A staining (Fig. 5A) suggests it is possible that when RSPH1 is depleted, other non-assembled radial spoke head components being still produced in the cell might accumulate outside of the cilia axoneme compartment. A slight cytoplasmic accumulation of ROPN1L can also be observed in RSPH1-deficient cells indicating there could also be some level of impeded assembly of spoke stalk components as well (Fig. 6A), however this is much less marked.

Finally, we also used two well-established diagnostic markers of axoneme integrity DNAH5 and DNALI1 to confirm that defects in RSPH1 do not affect the dynein arm based motors, as was suggested by the TEM analysis showing their apparent retention in *RSPH1*-mutated cilia. DNAH5 and DNALI1 antibodies specifically stain the outer and IDA, respectively, and we confirmed that the ODAs and IDAs were not perturbed as a consequence of *RSPH1* mutations since the staining pattern was similar in affected individuals compared with controls (Fig. 6B and C).

## DISCUSSION

Here we have shown that mutations in *RSPH1* are associated with PCD characterized by deficiency and loss solely of the central microtubular apparatus from cilia. *RSPH1* is the homolog of *Chlamydomonas* RSP1, encoding a radial spoke ‘head’ protein, and it is the third radial spoke head gene in which PCD-causing mutations have been identified, in addition to *RSPH4A* and *RSPH9*. Individuals with *RSPH1, RSPH4A* and *RSPH9* mutations represent a subtype of disease that has not been associated with disturbances of left–right body patterning (20,25,29). We report immunofluorescence analysis showing that *RSPH1*-mutated cilia lack RSPH1 protein, as well as the other radial spoke head components RSPH4A and RSPH9, but not the spoke stalk protein ROPN1L. The major ciliary MORN motif protein RSPH1 is therefore critical for the assembly of the radial spoke head, and *RSPH1*-mutated cilia are likely to lack the entire spoke ‘head’, but to retain the spoke ‘stalk’ structures (40). It is not easily possible to visualize the spoke heads in clinical TEM images from PCD patients, however *Chlamydomonas* mutant strains deficient for the homologous radial spoke proteins display reduction/loss of the entire head domain of the radial spokes whilst the radial spoke stalks are preserved (40,41).

We have investigated the underlying molecular basis of radial spoke head-deficient PCD disease in order to determine the reasons for CP loss. TEM analysis in longitudinal section identified transposition defects in *RSPH1*-mutated cilia, with absent CP and a peripheral microtubule doublet transposed into the space where the CP would normally be, towards the distal half of the cilia. We also discovered that an intermittent-appearing remnant CP can sometimes be seen, located proximally to the transposition event, towards the base of the cilia. Alternatively, because this was rarely possible to capture, this space is mostly seen as ‘empty’ with an apparent short remnant CP ‘stub’ sometimes visible at the cilia base. It seems likely that this loss, inability to extend, or partial retention of the CPr could result from a lack of its normal radial tethering within the axoneme (as provided by the radial spoke heads). Loss of the spoke heads, and a relative loosening of the central microtubules from their normal spokehead-directed radially-tethered confines could potentially allow them greater movement within the axoneme with a consequent loss of stability (or inability to form at all), such that the CP rotates in and out of the plane of the TEM section giving the intermittent appearance we captured.

We have observed this type of intermittent CP pattern before in *RSPH9*- (and *RSPH4A*-, unpublished data) mutated cilia, when these other radial spoke head proteins are deficient (25). We and others have previously reported that PCD-causing mutations in *RSPH4A* and *RSPH9* cause similar ciliary ultrastructural and motility defects (25,42,43). This provides evidence of a common molecular basis for the CP loss/transposition defects that result from mutations in any of the *RSPH1, RSPH4A* or *RSPH9* genes. In radial spoke head-deficient cilia, by inference from studies in *Chlamydomonas*, a disconnect between the radial spokes and CP would explain the replacement of the normal planar waveform (propelling the cilia forwards and backwards within the same plane) by the apparent circular movement that was recorded here in *RSPH1*-deficient cilia and elsewhere in *RSPH4A*- and *RSPH9*-mutated cilia (13,25,42). Studies of non-planar (circular) embryonic node cilia indicate that planar motion is directed by the presence of a CP (7). Our results show that RSPH1 and radial spoke head function is essential for the control of the ciliary waveform, to create the necessary beating pattern and force required for effective mucociliary clearance and other axonemal motility functions in the body.

Notably in *Chlamydomonas,* mutations in radial spoke head proteins results in dysmotility with loss of the head (but not stalk) domain of the radial spokes, however there is no accompanying loss of the CP (40,41). This different from the effect of the equivalent mutations underlying human PCD shown here. *Chlamydomonas* flagella have a characteristic asymmetric ‘breast stroke’ waveform that is quite distinct from the single-plane ‘whiplash’ pattern of human cilia motility, and the CP has significantly more movement within its axoneme compared with humans (44,45). The cross-species difference in CP retention upon mutation of the spoke heads is likely to reflect these differences in local control of axonemal waveform kinetics, with different resultant stresses on the CP affecting their formation and/or preservation differently.

This study has demonstrated that sequencing of a candidate NGS gene panel can identify PCD-causing mutations, and can distinguish between CP-deficient disease subtypes arising from *RSPH1*, *RSPH4A* and *RSPH9* mutations. CP loss/transposition defects account for up to 28% of PCD and these can be difficult to diagnose clinically, since affected individuals have normal laterality and cilia beat frequency around the normal range. Careful viewing of high-speed video is also required to fully appreciate the reduced amplitude and circling beat of their cilia. Our results can help in diagnosis of these difficult cases, showing that ∼20% of their cilia will be visible as either 9 + 0 or 8 + 1 arrangement. The presence of intermittent CP is, in our experience, unique to *RSPH1-*, *RSPH4A-* and *RSPH9*-mutated cilia deficient for radial spoke head mutations. This is distinguished from *HYDIN*-associated CP defects which display only very occasional absence of the CP. Since the clinical and molecular phenotypes conferred by mutations in any of the three radial spoke head genes *RSPH1*, *RSPH4A* and *RSPH9* appear to be broadly indistinguishable, genetic testing presents the only method to distinguish between these different defects.

The future application of this type of NGS technology will be in continuing to provide increasingly more comprehensive diagnostic testing for PCD, similarly to the progress shown here in development of diagnostic gene panels, compared with current methodologies available through UK diagnostic laboratories. It will facilitate in defining the PCD mutational spectrum across different populations, thereby increasing our current knowledge of the genetic architecture underlying PCD and the overall medical, health economic and societal impact of the disease in the UK and beyond. Broadening the availability of genetic testing will benefit affected families with PCD, primarily in promoting more effective and earlier diagnosis which has clearly been linked to improved disease outcomes (46), and also in assisting more rapid and informed genetic counseling for affected families.

## MATERIALS AND METHODS

### Subjects and ethics statement

Criteria for inclusion to the study were a diagnosis of PCD based upon documentation of typical symptoms including neonatal respiratory distress and chronic respiratory symptoms including rhinosinusitis, bacterial infections and fluid congestion, otitis media, bronchiectasis. Clinical tests were also documented in the cohort comprising light and electron microscopy findings showing ciliary motility and structural defects, and reduced nasal nitric oxide levels. Genetic studies were approved by the ethical committees of the Institute of Child Health/Great Ormond Street Hospital (#08/H0713/82) and the Comité de Protection des Personnes Ouest IV and Sud-Est II. Studies of unaffected individuals were separately approved by the University College London Research Ethics Committee (3187/001). Informed consent was obtained from all participants prior to history recording, blood drawing and nasal biopsy.

### NGS replication gene panel sequencing, alignment, variant detection and annotation

Whole genome amplified DNA (∼2 μg) was fragmented to an average size of 150 bp and subjected to library creation using established Illumina paired-end protocols (Illumina Inc., USA). Adapter-ligated libraries were amplified and indexed via PCR. A portion of each library was used to create an equimolar pool comprising 16 indexed libraries. Each pool was hybridized to SureSelect RNA baits and sequence targets were captured and amplified in accordance with the manufacturer's recommendations. Enriched libraries were subjected to 75 base paired-end sequencing (HiSeq 2000; Illumina). For the bait design process the SureSelect Target Enrichment protocol was used (Agilent SureDesign wizard, Agilent Technologies, USA), and probes were selected to target the coding exons of genes based on their Ensembl canonical ID, or by manually defining probe target regions where this failed. The NGS gene panel was processed at the Wellcome Trust Sanger Institute (Cambridge, UK) as part of the UK10K Project. The 150 PCD candidate genes included to the screen formed part of a larger collaborative project covering many disease candidate genes, such that in total there were 1223 gene targets comprising 19897 chromosomal regions, with a total region size of 3.348 Mb. This resolved to 50107 probes covering 4.512 Mb with 100% target coverage. The probe tiling parameters used a tiling density of 1×, no masking and balanced boosting.

The BWA alignment tool (47) was used to map sequence reads back to the genome (human reference hg19). To process the alignments and identify variations (SNPs and indels) we used both SAMtools mpileup (0.1.17) (48) and GATK UnifedGenotyper (1.3–21) (49), restricted to bait regions plus or minus a 100 bp window. Various quality filters were applied to each of the callsets separately and calls were then merged, giving preference to GATK information when possible. The SNP-Effects (http://snpeff.sourceforge.net/) and ANNOVAR (50) programs were used to annotate variations. Variant calls were annotated with 1000 Genomes allele frequencies (http://www.1000genomes.org/) and dbSNP132 rs ID numbers (51). Ensembl Variant Effect Predictor v2.2 analysis applied against Ensembl 64 was used to add functional annotation including coding consequence predictions, SIFT, PolyPhen and Condel annotations, and GERP and Grantham Matrix scores. Filters were applied to the resulting candidate gene variant profiles using the EVAR software tool v.0.2.2 beta. Variants present in 1000 Genomes, NHLBI-ESP exome variant server (http://eversusgs.washington.edu/EVS/) and dbSNP132 with a minor allele frequency >0.01 were removed, then variants not consistent with autosomal recessive in-heritance (biallelic changes). Variants were finally screened for possible motile cilia function, and for their presence in the Cilia Proteome (30).

### Sanger sequencing

The *RSPH1* transcript ID is RefSeq NM_080860.2. Variants of interest were amplified by PCR using primers designed with the Primer 3 web application (http://frodo.wi.mit.edu/primer3). Sequences and PCR conditions are available upon request. Sequences were analyzed compared with controls using Sequencher software (Gene Codes Corporation, Ann Arbor, MI, USA).

### Electron microscopy and high-speed video light microscopy

Respiratory epithelial cells were obtained using a cytology brush or rhinoprobe from the inferior turbinate of participants and immediately analyzed for cilia beat frequency (CBF) and beat pattern at 37°C under light microscopy as described (28). Samples were fixed in glutaraldehyde as previously described and an ultrastructural defect of the cilia was confirmed by TEM on a minimum of 100 cross sections and longitudinal sections where available (52). Electron micrographs and CBF from cases with *RSPH1* mutations were reviewed by one person (A.S.).

### *In silico* modeling of protein structure

The protein sequences used were taken from Swissprot for RSPH1 (Q8WYR4), RSPH4A (Q5TD94) and RSPH9 (Q9H1X1). Sequence and comparative phylogeny analysis was performed using Clustal Omega (http://www.clustal.org/omega/). Structural models of human RSPH1, RSPH4A and RSPH9 were generated by comparing the output from SMART (53), Uniprot (http://www.uniprot.org/) and Pfam (http://pfam.sanger.ac.uk/) programs. The predicted number of MORN repeats varies between programs because the definition of these domains is weak, based on a few amino acid residues at conserved positions. Predictions can also use data about the secondary structure, the position and predicted repeat number, thus the repeat numbers of such motifs predicted by various programs can differ, some definitions being stricter than the others. As explained in Figure 1 legend, the Pfam consensus showing seven MORN repeats in RSPH1 may be preferred over Uniprot and SMART which each predict six of these MORNs with two non-overlapping. The three dimensional model was created using I-TASSER (54).

### Immunofluorescence microscopy

Respiratory epithelial cells obtained by nasal brush biopsy from affected participants and controls were suspended in cell culture medium. Samples were spread onto glass slides, air dried and stored at −80°C until use. Cells were fixed with 4% paraformaldehyde for 4 min at room temperature, washed five times with phosphate buffered saline (PBS) and then permeabilized with 0.5% Triton X-100 for 10 min. After five more PBS washes, cells were incubated with 5% bovine serum albumin (Sigma-Aldrich, Dorset, UK) in PBS for 1 h. The cells were then incubated with primary antibodies overnight at room temperature using the following dilutions: axoneme-specific acetylated-α-tubulin monoclonal mouse antibody (Sigma T6793 6-11B-1, diluted 1:1000) to stain the entire axoneme, and rabbit polyclonal antibodies against RSPH1 (HP017382, Sigma, 1:100), RSPH9 (HPA031703, Sigma, 1:200), RSPH4A (HPA031196, Sigma, 1:75) and ROPN1L (HPA039193, Sigma, 1:100), to detect radial spoke proteins. Antibodies to DNAH5 (HPA037470, Sigma, 1:200) and DNALI1 (HPA028305, Sigma, 1:200) were used to confirm outer and IDA integrity, respectively. After five washes with PBS, cells were incubated with secondary anti-rabbit antibody (Alexa Fluor 488 Molecular Probes; Life Technologies, Paisley, UK) and secondary anti-mouse antibody (Alexa Fluor 594 Molecular Probes; Invitrogen) used at 1:1000 dilution. DNA was stained using DAPI (Life Technologies, Paisley, UK). Cells were finally washed five times with PBS, mounted in Vectashield (Vector Laboratories Ltd., Peterborough, UK) and confocal images were taken using a Zeiss LSM 710 (Zeiss Ltd., Cambridge, UK).

## SUPPLEMENTARY MATERIAL

Supplementary Material is available at *HMG* online.

## FUNDING

Funding for UK10K was provided by the Wellcome Trust under award WT091310. G.J. is supported by a stipend from Association Française Contre les Myopathies. M.S. is supported by an Action Medical Research UK Clinical Training Fellowship (RTF-1411), and M.S. and P.L.B. acknowledge funding from the Dutch Kidney Foundation (CP11.18). P.L.B. is a Wellcome Trust Senior Research Fellow, and acknowledges funding from the European Community's Seventh Framework Programme FP7/2009 under grant agreement no: 241955, SYSCILIA. P.L.B. and H.M.M. are supported by the Great Ormond Street Hospital Children's Charity*.* E.M.K.C. and H.M.M. are supported by grants from the Milena Carvajal Pro-Kartagener Foundation, Action Medical Research (GN2101) and Newlife Foundation for Disabled Children UK (10-11/15). Funding to pay the Open Access publication charges for this article was provided by the Wellcome Trust.

## Supplementary Material

Supplementary Data
