# Supplementary material for: Targeted NGS gene panel identifies mutations in RSPH1 causing primary ciliary dyskinesia and a common mechanism for ciliary central pair agenesis due to radial spoke defects
Source: Hum Mol Genet. 2014 Feb 11;23(13):3362–74. doi: 10.1093/hmg/ddu046 (PMC4049301; doi:10.1093/hmg/ddu046)
Supplement: Supplementary Data [file supp_23_13_3362__index.html]

Targeted NGS gene panel identifies mutations in RSPH1 causing primary ciliary dyskinesia and a common mechanism for ciliary central-pair agenesis due to radial spoke defects — Targeted NGS gene panel identifies mutations in RSPH1 causing primary ciliary dyskinesia and a common mechanism for ciliary central pair agenesis due to radial spoke defects — Targeted NGS gene panel identifies mutations in RSPH1 causing primary ciliary dyskinesia and a common mechanism for ciliary central pair agenesis due to radial spoke defects — Supplementary Data 

# Targeted NGS gene panel identifies mutations in *RSPH1* causing primary ciliary dyskinesia and a common mechanism for ciliary central pair agenesis due to radial spoke defects

## Supplementary Data

Supplementary Data

**Files in this Data Supplement:**

- Supplementary Data - Docx file
- Supplementary Video 1 - avi file
- Supplementary Video 2 - avi file
- Supplementary Video 3 - avi file
- Supplementary Video 4 - avi file
- Supplementary Video 5 - avi file
