# Supplementary material for: Targeted NGS gene panel identifies mutations in RSPH1 causing primary ciliary dyskinesia and a common mechanism for ciliary central pair agenesis due to radial spoke defects
Source: Hum Mol Genet. 2014 Feb 11;23(13):3362–74. doi: 10.1093/hmg/ddu046 (PMC4049301; doi:10.1093/hmg/ddu046)
Supplement: Supplementary Data [file supp_ddu046_ddu046supp.docx]

**SUPPLEMENTAL DATA**

**Supplementary video S1**

UCL-166 II:1 nasal biopsy cilia side view

**Supplementary video S2**

UCL-166 II:1 nasal biopsy cilia top view

**Supplementary video S3**

PCD-282 III:1 nasal biopsy cilia side view

**Supplementary video S4**

PCD-282 III:1 nasal biopsy cilia top view

**Supplementary video S5**

Unaffected control cilia side view
